# Supplementary material for: Unusual Association of NF-κB Components in Tumor-Associated Macrophages (TAMs) Promotes HSPG2-Mediated Immune-Escaping Mechanism in Breast Cancer
Source: Int J Mol Sci. 2022 Jul 18;23(14):7902. doi: 10.3390/ijms23147902 (PMC9324337; doi:10.3390/ijms23147902)
Supplement: Supplementary file 1 [file ijms-23-07902-s001.zip › ijms-1819739-supplementary.pdf]

Supplementary Material

Unusual association of NF-kB components in tumor associated macrophages (TAMs) promotes HSPG2-mediated immune-escaping mechanism in breast cancer

Veronica De Paolis[1], Fabio Maiullari[2,3], Maila Chirivì[4,5], Marika Milan[2,5], Chiara Cordiglieri[2], Francesca Pagano[1], Alessandra Rita La Manna[6], Elena De Falco[7,8] ,Claudia Bearzi[2,9], Roberto Rizzi[2,7]* and Chiara Parisi[1]*

1 Institute of Biochemistry and Cell Biology, National Research Council of Italy (IBBC-CNR), Via Ercole Ramarini, 32, Monterotondo, 00015 Rome, Italy; veronica.depaolis@ibbc.cnr.it (V.D.P.); francesca.pagano@cnr.it (F.P.)
2 Fondazione Istituto Nazionale di Genetica Molecolare, Via F. Sforza, 35, 20122 Milan, Italy; maiullari@ingm.org (F.M.); marika.milan@policlinico.mi.it (M.M.); cordiglieri@ingm.org (C.C.); claudia.bearzi@itb.cnr.it (C.B.)
3 PhD Program in Cellular and Molecular Biology, Department of Biology, University of Rome "Tor Vergata", Via della Ricerca Scientifica, 1, 00133 Rome, Italy
4 Department of Molecular Medicine Sapienza University, Viale Regina Elena, 324, 00161 Rome, Italy; maila.chirivi@uniroma1.it
5 UOC Neurology, Fondazione Ca'Granda, Ospedale Maggiore Policlinico, Via F. Sforza, 28, 20122 Milan, Italy
6 Breast Unit, Department of General Surgery, Santa Maria Goretti Hospital, via Guido Reni snc, Azienda Unità Sanitaria Locale (AUSL), 04100 Latina, Italy
7 Department of Medical Surgical Sciences and Biotechnologies, Sapienza University of Rome, C.so della Repubblica, 79, 04100 Latina, Italy; elena.defalco@uniroma1.it
8 Clinica Mediterranea Cardiocentro, Via Orazio, 2, 80122 Napoli, Italy
9 Institute for Biomedical Technologies, National Research Council, Via Fratelli Cervi, 93, 20054 Segrate, Italy
* Correspondence: roberto.rizzi@uniroma1.it (R.R.); chiara.parisi@cnr.it (C.P.)
† These authors equally contributed to this work.

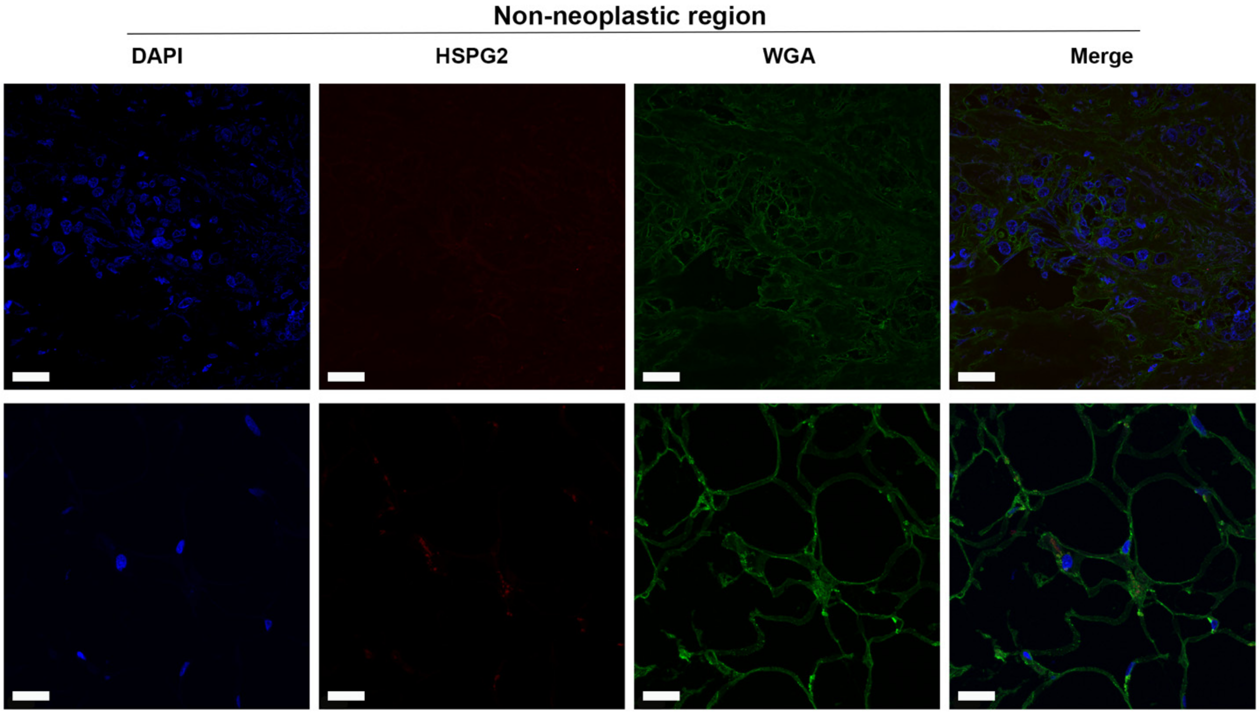

Supplementary Figure S1. HSPG2 staining in non-neoplastic region. Immunofluorescence analysis of HSPG2 was performed in the surrounding non-neoplastic region of TNBC biopsies. HSPG2 was stained in red, while tissue section was detected in green with WGA. Nuclei were counterstained with Dapi. Scale bars represent 40 µm. The representative images derived from qualitative analysis of TNBC biopsies of n = 3 patients.
